# Signal Processing Using a Circular Sensor Array to Measure the Torsional Angle of a Bolted Joint

**DOI:** 10.3390/s24092719

**Published:** 2024-04-24

**Authors:** Thorben Schüthe, Karl-Ragmar Riemschneider, Andreas Meyer-Eschenbach

**Affiliations:** Faculty of Engineering and Computer Science, Hamburg University of Applied Sciences, 20099 Hamburg, Germany; karl-ragmar.riemschneider@haw-hamburg.de (K.-R.R.); andreas.meyer-eschenbach@haw-hamburg.de (A.M.-E.)

**Keywords:** magnetic angle measurement, magnetic resolver, circular sensor array, torsion measurement, bolted joint torsional stress, bolt preload monitoring, Gaussian process regression

## Abstract

This study presents a new approach to determining the preload force of bolted joints. The concept involves measuring the torsional angle without contact. For this purpose, we present a circular magnetic sensor array integrated into the torque wrench. The torsional angle in bolted joints depends on the dimensions of the screw and the materials used and is typically less than four degrees. For this reason, one requirement is a high angular resolution so that a continuous recording of the torsion angle is feasible during the assembly process. This can be achieved using the circular sensor array and adapted signal processing methods. Two signal processing approaches are utilized. First, the direct method uses the discrete Fourier transformation to calculate the rotation angle from the signal phase. This approach is robust to signal distortion and does not depend on signal amplitude. Second, the method with a learning phase employs Gaussian process regression to minimize the angle error. In an experiment, both approaches were applied within a test bench and showed promising results. The direct method demonstrated a very good angular resolution without training and calibration. For mobile and less-complex applications where a reference system is unavailable, the direct method is preferable. However, in complex measurement systems where reference systems can be utilized initially, significant enhancements to an excellent resolution can be achieved through prior training.

## 1. Introduction

Digital torque wrenches are widely employed for the precise tightening of bolted joint connections [1,2]. These devices not only measure the torque applied but also often provide information regarding the angle of rotation during the assembly process. The combination of torque and angle plays a crucial role in ensuring the accuracy and integrity of the bolted joints. Knowledge of the preload force $F_{M}$, which holds the workpieces together, is of particular interest here. Measuring the torque is state-of-the-art, but not completely precise for determination of the preload force. This is because the friction on the bolt has a major influence. In a typical assembly application, the friction can only be estimated approximately. This can be described using Equation (1) for the tightening torque $M_{A}$. This torque is calculated by the thread torque $M_{G}$ and the head friction moment $M_{K}$.

(1) $$\begin{array}{cc} M_{A} & =M_{G}+M_{K} \end{array}$$

Each of the two moments $M_{G}$ and $M_{K}$ contains the preload force and known parameters of the bolt. Both moments also have an individual coefficient of friction $\mu_{G}$ and $\mu_{K}$, which depends on the surface properties. The aim is to neglect the head friction $\mu_{K}$ when calculating the preload force by measuring the torsion angle of the bolt during assembly. In this study, we propose a new approach based on torsional angle measurement. The solution utilizes modern magnetic sensing technologies and signal processing methods.

**Figure 1:**
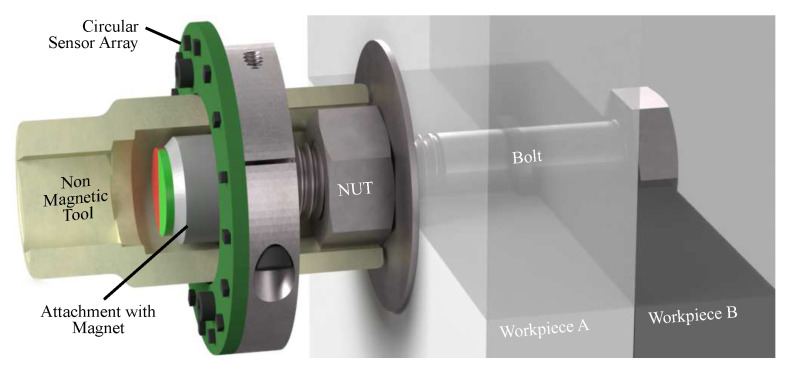
Tool with circular magnetic sensor array to fasten a bolted joint. A magnetic encoder is placed at the thread of the bolt. This design was first presented in a patent application [7].

Recently, a new method for the noncontact measurement of torsion in bolted joints using a square magnetic sensor array was presented in [3], in which the recorded data provide valuable insights for monitoring the assembly process. In this method, a magnet is positioned at the top of the bolt to generate the magnetic field for the sensor array. However, when the magnet is centrally located above the squared arrangement, the outer sensors do not detect the same magnetic field strength as the sensors in the center. Consequently, this discrepancy leads to different operating points of individual sensors within the array. To address this issue, a circular array can be employed so that all sensors detect nearly the same field strength. The circular arrangement also supports the design constraints of a tool, as shown in Figure 1. By placing the magnet inside the socket, the angle of the assembly can be directly measured on the workpiece, eliminating the need for inertial angle measurement within the electronic torque wrench. The inertial or gyroscopic sensor may introduce remarkable deviations in cases where the torque wrench is tilted or improperly operated. On the other hand, the magnetic angle measurement has to be very precise. The required accuracy is well within one degree and, therefore, outside the specifications of commercially available magnetic sensors [4,5,6].

## 2. State of the Art

Magnetic sensors are commonly used to measure rotational speed or the absolute angle of rotation. The rotational speed sensor operates incrementally using a passive or active encoder. The latter type of sensor is often used for axial measurement of the angle on a shaft, whereby a diametrically magnetized encoder magnet at the end of the shaft provides the magnetic field. A patent application has proposed an axially positioned sensor within a specialized tool for direct angle measurement [8]. This approach requires a very precise sensor and an accurate arrangement of the sensor and magnet.

Measuring the absolute angle in radial position on the side of the shaft is also possible with magnetic sensors. However, radially operating methods require signal calibration to obtain accurate results [9,10]. Many of these systems are based on linear Hall sensors. Special encoder shapes have been proposed to avoid calibration [11,12,13]. Another approach to measuring the radial angle involves using magnetic encoders with two differently magnetized strips [14].

Furthermore, for the torsion measurement in bolted joints, a previous work [3] proposed a squared sensor array. Nevertheless, for the design of an automatic measuring assembly tool for screw fastening, a circular sensor array is more suitable. Similar designs in the field of current measurement utilize arrays with anisotropic magnetoresistive (AMR) sensors [15] or tunnel magnetoresistive (TMR) sensors [16,17]. A distinct advantage frequently highlighted in the literature is the geometric symmetry offered by circular sensor arrays, which enables two opposing sensors to compensate for their deviations from the rotation axis.

## 3. Hardware Description

This section explains the sensor function, hardware arrangement, signals, and data acquisition.

### 3.1. Sensor and Magnet Arrangement

**Figure 2:**
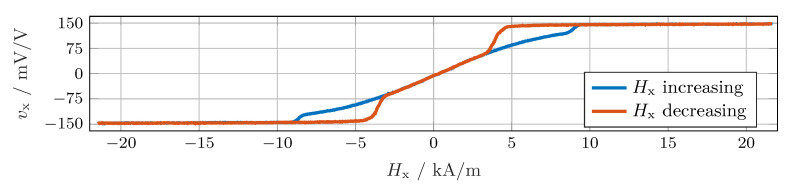
Characteristic curve of a TMR sensor for an increase in field strength (blue) and a subsequent decrease (red). An automated setup for characterizing the angle sensors was presented in [22].

Sensors based on the TMR effect are suitable for the construction of sensor arrays because of their remarkable sensitivity and miniaturization capabilities [18,19,20,21]. The design of the circular sensor array uses TMR sensors that can operate in both the linear and saturated ranges [5,22,23]. This behavior is illustrated with the characteristic curve in Figure 2. In addition to the ranges described, another dependency on the course of the field strength is present. The decisive factor here is whether the field strength increases or decreases. The dissertation by Wurft [24] details the physical effects on the sensitive elements. The main influences are the dimension, shape, and material of the magnetic tunnel junction [25]. The magnet and angle sensor are usually located on the central rotation axis. The intended application for a screw assembly tool requires a different measuring arrangement because the axial position is not available. Instead, the sensors are shifted radially and placed lateral to the magnet.

**Figure 3:**
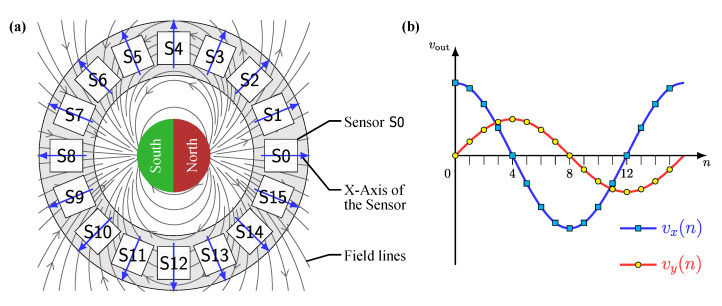
(**a**) Illustration of the field lines and circular sensor array composed of N=16 sensors S0–S15. The blue arrows represent the rotated alignment of the sensors around their *x*-axis. (**b**) Output signals $v_{x}\left(n\right)$ and $v_{y}\left(n\right)$ as functions of sensor location, where *n* is the sensor index 0–15.

For this reason, the magnetic field to be measured is considered separately at each sensor position. Figure 3a presents a schematic illustration of the output signals generated by a circular sensor array. Each sensor consists of two Wheatstone bridges arranged orthogonally. As an output, the bridges supply two differential voltages $v_{x}$ and $v_{y}$. The sensors are positioned along a circle and rotated according to their angle, as indicated by the blue arrows. In the center of the array, the *x*-axis aligns with the axis of the magnet.

In the circular array configuration, the sensors are arranged at a stepwise rotating angle, resulting in a cosine wave when the output signals $v_{x}\left(n\right)$ of all sensors are plotted over their index *n* in the array, as shown in Figure 3b. The same applies to the sine shape of the output signal $v_{y}\left(n\right)$. With the radial magnetization and magnet position, the maximum $v_{x}\left(n\right)$ occurs at sensor S0, and the minimum $v_{x}\left(n\right)$ is at S8. For the voltage $v_{y}\left(n\right)$, these occur at sensors S4 and S12, respectively. With the rotation of the magnet, these sensor indexes change accordingly. However, the two voltage curves have different amplitudes. The difference can be explained with the magnetic dipole equation [26], considering only the $H_{x}$ component, which represents the field strength along the *x*-axis.

(2) $$H_{x}\left(r\right)=\frac{1}{4\;\pi }\;\left(\frac{3\;r_{x}\;\left(m^{T}\;r\right)}{r^{5}}-\frac{m_{x}}{r^{3}}\right)$$

where, the distance from the origin to the sensor $r_{s}$ and the distance from origin to the dipole $r_{d}$ result in a vector $r=r_{d}-r_{s}={\left(r_{x}\;r_{y}\;r_{z}\right)}^{⊺}$ with length *r*. The unit of all distances are meter (m), the unit of field strength $H_{x}$ is ampere per meter (A/m), and the magnetization ampere times square meter is A^2^. With the magnetization of $m={\left(1\;0\;0\right)}^{⊺}$, the field strength at sensor S0 has a value of $H_{x}=\pi /2$. For sensor S4, the field strength is $H_{x}=\pi /4$.

When the direction of the magnetization vector and the detected field component in this equation align, the resulting field strength is at its maximum. When these two vectors are orthogonal, a decreased field strength is detected. The amplitude is further influenced when the plane of the circular array and the plane of the magnet differ. Then, the third power of the geometric distance *r* between the magnet and the sensor reduces the field strength components and voltages $v_{x}\left(n\right)$ and $v_{y}\left(n\right)$.

However, this ideal behavior only applies if the field strength is in the linear range of the sensor characteristics; see Figure 3. In practice, deviations from these ideal conditions must be assumed.

### 3.2. Data Acquisition

**Figure 4:**
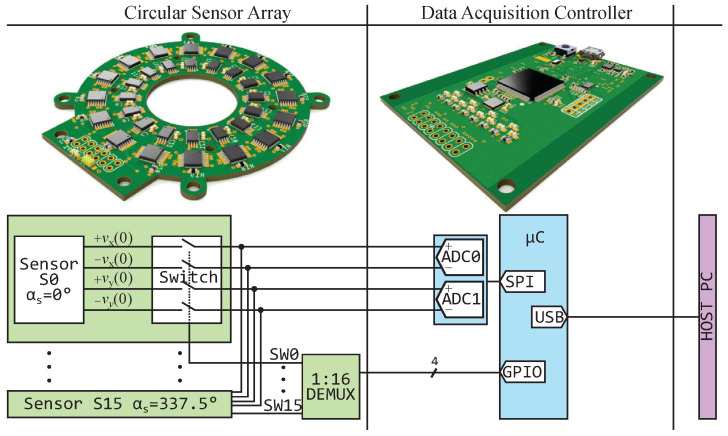
Block schematic of the sensor array control circuit. The array and the data acquisition controller are separated such that no magnetic flux guidance from the parts on the PCB (printed circuit board) acquisition influences the measurement.

The implemented ring array consists of N=16 sensors [5]. Each sensor has a rotated angular arrangement of angle $\alpha_{s}\left(n\right)=2\;\pi \;\left(N-1\right)/n$. The differential sensor signals $v_{x}\left(n\right)$ and $v_{y}\left(n\right)$ are fed through an analog switch [27]. To sample all the sensors in sequence, a demultiplexer is used [28]. Thus, the four signals of a single sensor are converted simultaneously. The conversion is conducted with two simultaneous sampling 16 bit analog-to-digital converters (ADCs) with differential input and a maximum sampling rate of 4 MSPS [29]. The multiplexer and the ADC are connected to a microcontroller [30]. Its USB interface allows communication with a host computer with a maximum of 460 Mbits. Figure 4 shows a schematic of this setup.

## 4. Signal Processing

This section describes two signal processing methods for the circular sensor array. The first is a direct method for calculating angles, and the second describes a training method.

### 4.1. Direct Method

The direct method for angle calculation evaluates the phase shift of the sensor array signals using the discrete Fourier transform from Equation (3). The same nomenclature applies as before, where index *n* stands for a sensor of the array having N sensors.

(3) $$V_{x}\left(k\right)=\sum_{n=0}^{N-1}v_{x}\left(n\right)\;\exp\left(\;-j\;2\;\pi \;\frac{k\;n}{N}\right),\;\;k=\left(0,\ldots ,K-1\right)$$

Because of the angular positions of the sensors within the circular arrangement, signal $v_{x}$ forms a sinusoidal wave with an angular position frequency of one. The phase of this frequency is influenced by the rotation of the magnet. Since only the first spectral line is used, the calculation of the other lines in the sum of Equation (3) can be omitted. The angle of rotation is calculated using Equation (4):

(4) αx=∠Vx(1)=atanImVx(1)ReVx(1)

The same procedure can be used to calculate the angle via $V_{y}$. To determine the mean value $\alpha_{s}$ of both angles, the phase of $V_{y}$ must be added to π/2 before calculating the phase angle:

(5) αs=12αx+atanImVy(1)ReVy(1)+π2

### 4.2. Training Method

**Figure 5:**
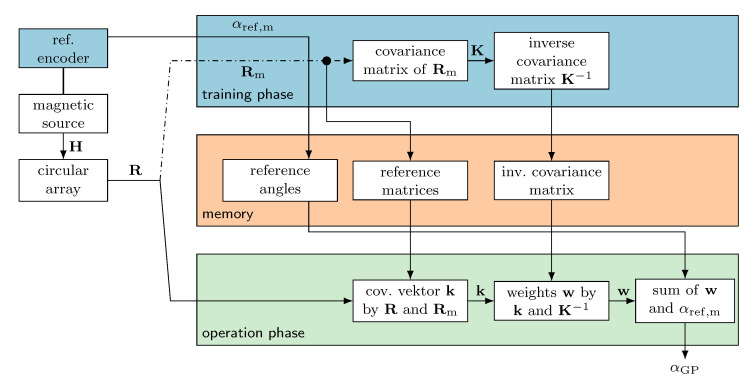
Flow chart for the determination of the absolute angle $\alpha_{\mathrm{GP}}$ using Gaussian process regression. The values from the training phase (blue) are stored in a nonvolatile memory (red). These values are required in the operation phase (green) to estimate the encoder angle from the current measured values and the reference values by weighted sums.

A machine learning method was used for training, with Gaussian process (GP) regression proving to be a well-suited approach. The procedure is outlined in [31] for a squared sensor array for angular measurement. We adapted this method for the circular sensor array. In general, the GP regression consists of two phases, as overviewed in Figure 5.

Within the training phase, a limited number $N_{\mathrm{ref}}$ of measurements are collected together in a matrix $R_{m}={\left(v_{x}\;\;v_{y}\right)}^{⊺}$ with reference angles $\alpha_{\mathrm{ref}}$. These data are needed to calculate the covariance matrix K with the elements $K_{mn}=\mathrm{cov}\left(R_{n},R_{m}\right)$. In this study, the quadratic exponential function is used as the covariance function in the following form:

(6) $$\begin{array}{c} \mathrm{cov}\left(A,B\right):=\exp\left(\frac{||A-{B||}_{F}^{2}}{2\;l^{2}}\right) \end{array}$$

where *l* acts as the length scale, which has to be optimized within the training phase through simulation or using further measurement values between the reference points [32].

For the operation phase, the measurements, reference angles, and covariance matrix are stored in a nonvolatile memory. Here, the covariance vector k is calculated using the covariance function of the stored reference values and the actual measured values R. The elements of the vector are given by $k_{m}=\mathrm{cov}\left(R,R_{m}\right)$. After this step, the weight vector w can be calculated:

(7) $$\begin{array}{c} w=k^{⊺}\;\cdot \;K^{1} \end{array}$$

The last step is the summation of the multiplication products of the weights and reference angle values.

## 5. Experiments

We employed a test bench for the experiments. The position of the field-generating magnet was specific to the application and located approximately on the central axis of the ring array. The output values of both a single sensor and the sensor array were analyzed. Finally, both signal processing methods described above were compared.

### 5.1. Test Bench for Verification

**Figure 6:**
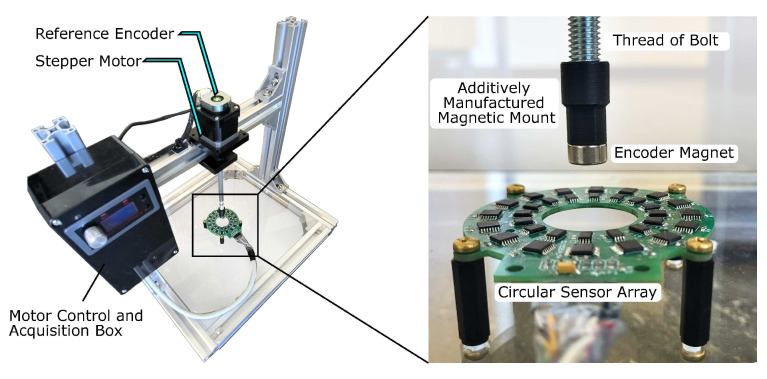
Test bench for the angular measurement at the head of a threaded bolt. The motor control and acquisition box contains the data acquisition controller, a stepper motor controller, and an interface for the reference encoder. The magnet is located above the circular sensor arrangement at the shaft end of a threaded bolt on an additively manufactured mount.

We examined the signal processing methods and the resulting angular errors on the test bench shown in Figure 6. The measurement data from the circular sensor array were recorded with the microcontroller system and evaluated on a PC. A capacitive reference encoder on the test bench provided the angular information to determine the angular accuracy of the magnetic measurement. The encoder had an absolute angular resolution of 14 bits, resulting in an accuracy of around 0.02° [33]. Considering the potential application of the torsion measurement in screws, an M8 × 160 bolt was employed as the shaft. At the end of the bolt, an attachment on the thread held a diametrically magnetized cylindrical magnet of grade N45. This magnet had a diameter of 10 mm and a width of 8 mm. A stepper motor rotated the shaft at a constant speed of 8 rpm during the entire measuring process.

### 5.2. Output Signals

**Figure 7:**
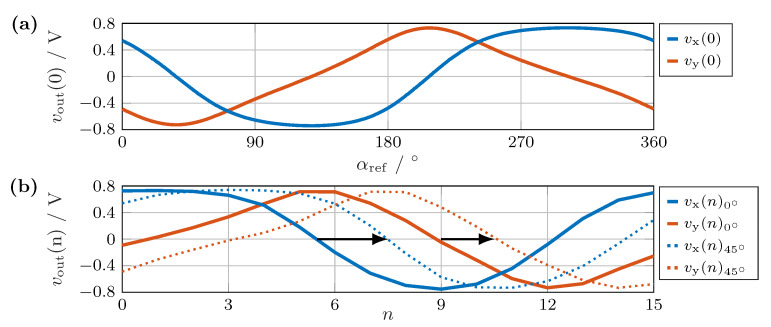
(**a**) Output signal of sensor S0 in the array over a full rotation of the magnet. (**b**) The output signals $v_{x}\left(n\right)$ (blue) and $v_{y}\left(n\right)$ (red) of the circular sensor array at an encoder angle of about 0° (solid) and at a rotation of 45° (dashed). The rotation leads to a shift, as indicated by the black arrows.

The magnet utilized in the experiment produced a field strength exceeding 15 kA/m at the sensors, leading to sensor saturation (see Figure 2). In the saturated range, the output signal was independent of the field strength, depending only on the field direction. Consequently, both signals reached their maximum output amplitude. When observing the curve over a complete rotation at sensor S0, as depicted in Figure 7a, we recognize a pronounced distortion due to the sensors operating in areas in which the sensor characteristic was completely or partially saturated. Therefore, the sensor voltages did not follow the ideal curve shown in Figure 3.

The saturation effects caused similar amplitudes of the two curves, as shown in Figure 7a. Figure 7b illustrates the curves obtained for the individual angular positions, ranging from S0 to S15. The phase shift induced by the rotation of the encoder magnet is clearly indicated.

### 5.3. Measurement Results

**Figure 8:**
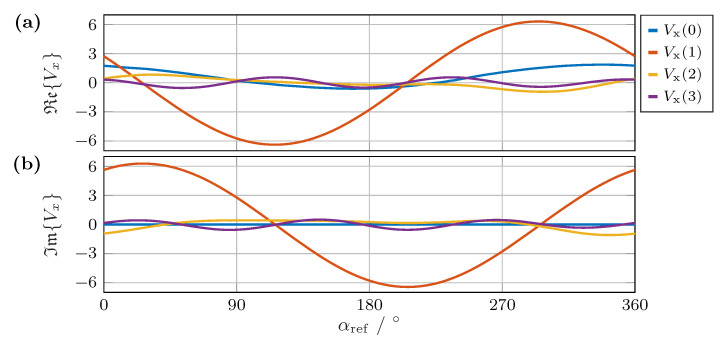
(**a**) Real and (**b**) imaginary parts of the first four frequencies of the Fourier transform of $v_{x}$ over a complete rotation of the encoder as a function of the reference angle.

First, we considered the result of the direct method using Fourier transformation. For this purpose, the angle between two vectors was defined as the error measure. In addition, we defined the zero point for the comparison. The results of the Fourier transformation of the output signals of $v_{x}$ over a full rotation of the magnet are shown in Figure 8a,b for the real and imaginary parts, respectively. The components of the first frequency $V_{x}\left(1\right)$ are shown in red. Based on these two curves of the real and imaginary parts of a signal, a single output sequence of the array was sufficient for the angle calculation.

**Figure 9:**
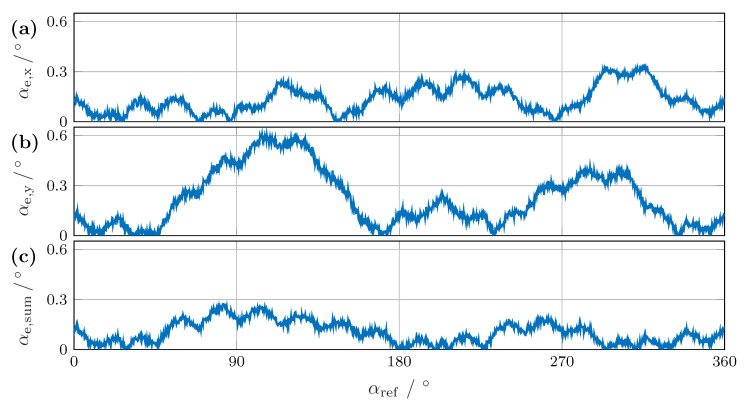
Angular error between the reference angle and the calculation using the Fourier transform. (**a**) Error $\alpha_{e,x}$ from signal $v_{x}$, (**b**) $\alpha_{e,y}$ from signal $v_{y}$, and (**c**) $\alpha_{e,s}$ from their sum.

Averaging the angles calculated from $v_{x}$ and $v_{y}$ leads to a higher resolution and may offer an error reduction. For comparison, the angular errors from the individual bridge signals—$\alpha_{e,x}$ for $v_{x}$, $\alpha_{e,y}$ for $v_{y}$, and $\alpha_{e,s}$ for the sum $\alpha_{s}$—are shown in Figure 9. The two individual calculations in Figure 9a,b had a larger angular error than their sum in Figure 9c. For $v_{x}$, the maximum error was about 0.3°, and for $v_{y}$ it was about 0.6°. After summing, the angular error for a full rotation was less than 0.3°. Notably, only the zero point was set, and no other calibration was carried out.

**Figure 10:**
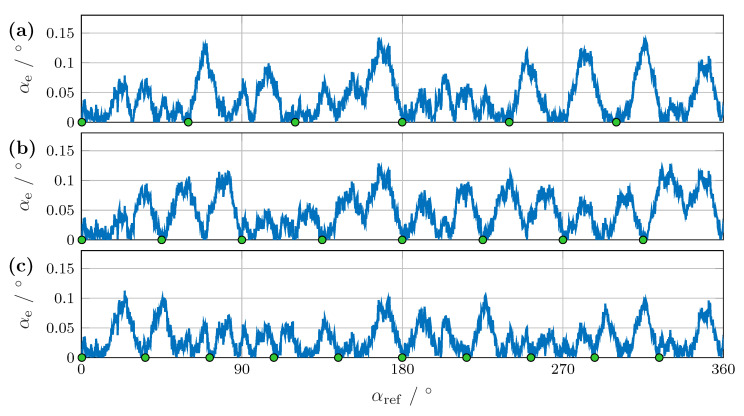
Difference between the reference angle and measured angle after training with Gaussian process regression with (**a**) six, (**b**) eight, and (**c**) ten reference points.

Next, we evaluated the angular error based on the calculation using GP regression. Figure 10 shows the angular error $\alpha_{e}$ for six, eight, and ten reference positions. With just six reference positions in Figure 10a, the angular error $\alpha_{e,6}$ was reduced to less than 0.15° over a full rotation. When further reference values were added, the peaks between the reference points were reduced, resulting in an error $\alpha_{e,10}$ of less than 0.12°, as shown in Figure 10c.

Table 1 shows the evaluation of the angular error with its maximum $\alpha_{e,\max}$, mean $\alpha_{e,\mathrm{mean}}$, and standard deviation $\alpha_{e,\mathrm{std}}$ for different numbers of references $N_{\mathrm{ref}}$ over a full rotation. With an increasing number of references, only slight improvements occur, and the length scaling *l* decreases. As this is an angle evaluation, small deviations in the input signals for the angle calculation had a significant impact on the output signal.

Each case (i.e., six, eight, or ten references) requires a parameter optimization of the length scaling *l*, which must be carried out in the training phase. However, more references lead to increased memory requirements and computation effort in the operation phase. Therefore, limiting the number of references is beneficial. Additional references can be omitted if no improvement is observed.

### 5.4. Future Tool Design

The results shown were carried out on a measuring setup to investigate the accuracy of the measuring system. The only component made of ferromagnetic material in the vicinity of the sensor array is the bolt on which the magnet is positioned. For further investigations, the influence of the socket on the results must first be checked. This should be positioned in the center of the array, as shown in Figure 1. The design and material of the socket should be taken into account in order to minimize the influence on the angle measurement. Materials with low ferromagnetic properties, such as bronze or titanium, would be preferred. However, this is not always practicable in the application or is associated with excessive costs, which is why steel alloys are used. The extent to which the magnetic field of the encoder is weakened in the center of the socket and whether a combination with the sensor array would be feasible must be examined. The arrangement ensures that the distance between the encoder and the circular sensor array is small. Therefore, the field strength at the sensors is in an advantageous measuring range. As the intensity of the magnetic field decreases with cubic distance, the influence of materials outside the array is typically negligible. It is also necessary to investigate how flux-carrying materials in the surrounding environment affect the measured values. It can be assumed that, due to the symmetry properties of the array, the effects mainly influence the field strength, but not the resulting angle values.

## 6. Application Perspectives

The proposed solution can be an alternative to the classic resolver based on the transformer principle with a rotating coil. Similar advantages are expected with regard to robustness in harsh environments, contactless operation, no mechanical wear and friction, and nonsensitivity to oil and dust. In addition, the energy requirement is low, and the dimensions can be adjusted to the individual application. The TMR sensors and the signal processing are well suited for an embedded microcontroller to control modern mechatronic applications.

In addition to the preload force measurement in bolted joints, the torsion measurement holds significance in various other scenarios. For instance, in a laboratory setting, a torsion test rig currently relies on conventional and highly precise angle encoders [34]. However, these encoders necessitate mechanical coupling and alignment. On the other hand, the proposed magnetic sensor arrays offer the advantage of measuring without a mechanical coupling.

For example, using the direct measurement method without training presents an opportunity for detecting torsional vibrations in shafts within drive trains operating at medium speeds. This application targets rotational speeds of up to 3000 rpm and requires an angular error of less than 0.2°.

Another relevant application is the utilization of precise angular measurements in rotational rheometers used for highly viscous materials [35]. Implementing a cost-effective inline rheometer can benefit from a circular magnetic sensor array. The initial reference values can be recorded in a training phase using a sophisticated calibration device for accurate measurements.

A potential application field pertains to observing the low dynamic backlash at the output shaft of a gearbox, particularly in situations with extremely high gear ratios (1:1000 or higher). The static reference can be obtained through long-term measurements conducted beforehand. An example of this application is the control loop of a large parabolic antenna drive, characterized by a high mass moment of inertia [36].

## 7. Conclusions

This study presents two signal processing methods for angle measurement utilizing circular magnetic sensor arrays: a direct method and a method involving a prior training phase.

The direct method demonstrated an accuracy of 0.3° or less without training and calibration. The discrete Fourier transform was employed to combine the sensor measurements and obtain the resulting angle. This approach is robust to signal distortion and does not depend on signal amplitude. By incorporating a training phase based on Gaussian process regression, the angular accuracy was further improved. With six reference values, the experimental results show a maximum error of 0.14°. For mobile and less-complex applications where a reference system is unavailable, the direct method is preferable. However, in complex measurement systems where reference systems can be utilized initially or continuously, significant enhancements can be achieved through prior training.

Future research should include the development of a complete tool and an optimization of the human interface of the controlling and analyzing software.

Overall, this work demonstrates the advantageous use of modern magnetic sensor technology combined with the principle of a sensor array. 

## Figures and Tables

**Table 1:** Results of the GP regression angle calculation for different numbers of reference values. The maximum, mean, and standard deviation of the angular error are given in degrees. Furthermore, the length scale *l* is given.

$N_{\mathrm{ref}}$	3	4	5	6	7	8	9	10	11	12
*l*	8.200	6.106	4.425	3.447	2.796	2.367	2.049	1.813	1.623	1.473
$\alpha_{e,\max}$	2.342	0.827	0.288	0.142	0.136	0.129	0.115	0.113	0.106	0.110
$\alpha_{e,\mathrm{mean}}$	1.319	0.392	0.098	0.040	0.038	0.046	0.034	0.032	0.029	0.033
$\alpha_{e,\mathrm{std}}$	0.647	0.208	0.072	0.033	0.029	0.031	0.025	0.025	0.021	0.025

## Data Availability

The raw data supporting the conclusions of this article will be made available by the authors on request.

## References

[1] SCS Concept (2018). Freedom 3 Torque/Angle DigitalWrench, 30 Nm. Datasheet. https://www.scsconcept.com/brochure/en/Freedom3.pdf.

[2] Meyer-Eschenbach A., Grätsch T., Höring G., Rajabi D., Knoth J.-H., Fischer M., Stahl M., Büchle J., Dieterle H., Brahm F. (2022). Schraubenverbindungen.

[3] Schüthe T., Riemschneider K.-R., Meyer-Eschenbach A. Magnetic sensor array for determining the assembly torsion and preload of a bolted joint. Proceedings of the 2022 IEEE Sensors Applications Symposium (SAS).

[4] NXP Semiconductors (2014). KMZ60 Angle Sensor with Integrated Amplifier. Datasheet. https://www.nxp.com/docs/en/data-sheet/KMZ60.pdf.

[5] TDK Electronics (2019). TAS2143-AAAA. Datasheet. https://product.tdk.com/system/files/dam/doc/product/sensor/angle/tmr-angle/data_sheet/ds_sensor_tmr-angle_tas2143-aaaa_en.pdf.

[6] Infineon (2019). TLE5501 Angle Sensor. Datasheet. https://www.infineon.com/cms/en/product/sensor/magnetic-sensors/magnetic-position-sensors/angle-sensors/tle5501-e0001/.

[7] Schüthe T., Meyer-Eschenbach A., Riemschneider K.-R., Weithoff F., Richter S., Brodersen J. (2022). Werkzeug für die Schraubenmontage mit Magnetsensor-Array zur Torsionsmessung. Patent Application.

[8] Hongde C., Hongwu Z., Rongxiang Z., Wen F. (2019). Intelligent Fastener Capable of Sensing Looseness of Fastener Nut and Online Monitoring System. Patent Application.

[9] Infineon (2019). TLE5xxx(D) Calibration 360°. Datasheet. https://www.infineon.com/dgdl/Infineon-TLE5xxx(D)_Calibration_360_AN-v02_00-AN-v02_00-EN.pdf:PDF?fileId=5546d46264a8de7e0164f09d8bfa228d.

[10] Sirohiwala A., Bussing W., Allegro MicroSystems Inc. (2015). Advanced On-Chip Linearization in the A1332 Angle Sensor IC. Datasheet. https://www.allegromicro.com/en/insights-and-innovations/technical-documents/hall-effect-sensor-ic-publications/advanced-on-chip-linearization-a1335-angle-sensor-ic.

[11] Jerance N., Arlot R. (2010). Positionssensor mit Veränderlicher Magnetisierungsrichtung und Produktionsverfahren. Patent.

[12] Schweizer P., Kenney C., Andrews R. (2020). Off-Axis Magnetic Angular Sensor Using a Magnetic Sensing Probe and Multipole Magnet. Array. Patent.

[13] Grönefeld M., Gerhartz J., Ohmer M. (2003). Verfahren zur Erfassung Linearer Relativbewegungen Zwischen Dauermagneten und. Sensoren. Patent.

[14] Bakos Á., Gozse I., Soumelidis A. Design and analysis of a magnetic off-axis rotary position sensing device. Proceedings of the 22nd Mediterranean Conference on Control and Automation.

[15] Grim V., Ripka P., Fischer J. Characterization of circular array current transducers. Proceedings of the IEEE Sensors Applications Symposium (SAS).

[16] Yu H., Qian Z., Liu H., Qu J. (2018). Circular array of magnetic sensors for current measurement: Analysis for error caused by position of conductor. Sensors.

[17] Guo C., Chen L., Zhang H. (2021). Current measurement for curved conductor based on 3-d coreless TMR sensor array. J. Phys. Conf. Ser..

[18] Süss D., Brückl H., Prügel K., Satz A., Raberg W. (2016). Magnetsensorbauelement und Magneterfassungsverfahren. Patent Application.

[19] Zimmer J., Satz A., Raberg W., Brückl H., Süss D. (2015). Device, Magnetic Sensor Device and Method. Patent Application.

[20] Paul J., Schnieders C., Traute J., Lehndorff R., Conca A., Leven B., Hillebrands B., Casper F., Jakob G., Kläui M. B3.2—Sensors Based on Tunnel Magnetoresistance—New Technology, New Opportunities. Proceedings of the SENSOR, AMA Service.

[21] Jin Z., Mohd Noor Sam M.A.I., Ando Y. (2021). Serial MTJ-based TMR sensors in bridge configuration for detection of fractured steel bar in magnetic flux leakage testing. Sensors.

[22] Schüthe T., Albounyan A., Riemschneider K. Two-Dimensional Characterization and Simplified Simulation Procedure for TMR Angle Sensors. Proceedings of the 2019 IEEE Sensors Applications Symposium (SAS).

[23] Wurft T., Raberg W., Prügl K., Satz A., Reiss G., Brückl H. (2019). Evolution of magnetic vortex formation in micron-sized disks. Appl. Phys. Lett..

[24] Wurft T. (2018). Investigation of the Magnetic Vortex State for Spin-Valve Sensors. Ph.D. Thesis.

[25] Suess D., Bachleitner-Hofmann A., Satz A., Weitensfelder H., Vogler C., Bruckner F., Abert C., Prügl K., Zimmer J., Huber C. (2017). Topologically Protected Vortex Structures to Realize Low-Noise Magnetic Sensors. arXiv.

[26] Lehner G., Kurz S. (2021). Elektromagnetische Feldtheorie.

[27] Nexperia B.V. (2021). 74HCT4066 Quad Single-Pole Single-Throw Analog Switch. Datasheet. https://assets.nexperia.com/documents/data-sheet/74HC_HCT4066.pdf.

[28] Nexperia B.V. (2021). 74HCT4067 16-Channel Analog Multiplexer/Demultiplexer. Datasheet. https://assets.nexperia.com/documents/data-sheet/74HC_HCT4067.pdf.

[29] Analog Devices, Inc. (2022). AD7380-4 Differential Input, Quad, External Reference Simultaneous Sampling, 16-Bit, SAR ADC. Datasheet. https://www.analog.com/media/en/technical-documentation/data-sheets/ad7380-4.pdf.

[30] Texas Instruments Inc. (2014). TM4C1294NCPDT 32-Bit-ARM-Cortex-M4F-Based MCU. Datasheet. https://www.ti.com/product/de-de/TM4C1294NCPDT.

[31] Schüthe T., Jünemann K., Riemschneider K.-R. Tolerance Compensation based on Gaussian Processes for Angle Measurements with Magnetic Sensor Arrays. Proceedings of the IEEE Sensors 2020.

[32] Rasmussen C.E., Williams C.K.I. (2006). Gaussian Processes for Machine Learning.

[33] CUI-Devices (2021). AMT212D-V Modular Absolute Encoder (14 Bit). Datasheet. https://www.cuidevices.com/product/motion/rotary-encoders/absolute/modular/amt212d-v.

[34] Dr. Johannes Heidenhain GmbH (2023). Angle Encoders Product Page. Datasheet. https://www.heidenhain.com/products/angle-encoders.

[35] Anton Paar GmbH (2023). Rheometer—The MCR Rheometer Series. Datasheet. https://www.anton-paar.com/uk-en/products/group/rheometer/.

[36] Ahmed M., Noor S.M., Hassan M.K., Soh A.C. (2014). A review of strategies for parabolic antenna control. Aust. J. Basic Appl. Sci..

